# Modeling of Cu(II) Adsorption from an Aqueous Solution Using an Artificial Neural Network (ANN)

**DOI:** 10.3390/molecules25143263

**Published:** 2020-07-17

**Authors:** Taimur Khan, Teh Sabariah Binti Abd Manan, Mohamed Hasnain Isa, Abdulnoor A.J. Ghanim, Salmia Beddu, Hisyam Jusoh, Muhammad Shahid Iqbal, Gebiaw T Ayele, Mohammed Saedi Jami

**Affiliations:** 1 Department of Civil Engineering, Faculty of Engineering, Najran University, P.O. Box 1988, King Abdulaziz Road, Najran 61441, Saudi Arabia; aaghanim@nu.edu.sa; 2 Civil and Environmental Engineering Department, Universiti Teknologi PETRONAS, 32610 Seri Iskandar, Perak Darul Ridzuan, Malaysia; 3 Institute of Tropical Biodiversity and Sustainable Development, Universiti Malaysia Terengganu, 21300 Kuala Terengganu, Malaysia; tehsabariah@umt.edu.my; 4 Civil Engineering Programme, Universiti Teknologi Brunei, Tungku Highway, Gadong BE1410, Brunei Darussalam; mohamed.isa@utb.edu.bn; 5 Department of Civil Engineering, Universiti Tenaga Nasional, Jalan Ikram-Uniten, 43000 Kajang, Selangor Darul Ehsan, Malaysia; salmia@uniten.edu.my; 6 Geo TriTech, No. 17, Persiaran Perdana 15A, Pinji Perdana, 31500 Lahat, Perak, Malaysia; mnhisyam.jusoh@gmail.com; 7 Department of Space Sciences, Institute of Space Technology, Islamabad 44000, Pakistan; shahidiqbal@outlook.com; 8 Australian Rivers Institute and School of Engineering, Griffith University, Nathan, QLD 4111, Australia; gebeyaw21@gmail.com; 9 Department of Biotechnology Engineering, Faculty of Engineering, International Islamic University Malaysia, P.O. Box 10, Kuala Lumpur 50728, Malaysia; saedi@iium.edu.my

**Keywords:** adsorption, artificial neural network, heavy metal removal, rice husk char

## Abstract

This research optimized the adsorption performance of rice husk char (RHC4) for copper (Cu(II)) from an aqueous solution. Various physicochemical analyses such as Fourier transform infrared spectroscopy (FTIR), field-emission scanning electron microscopy (FESEM), carbon, hydrogen, nitrogen, and sulfur (CHNS) analysis, Brunauer–Emmett–Teller (BET) surface area analysis, bulk density (g/mL), ash content (%), pH, and pH_ZPC_ were performed to determine the characteristics of RHC4. The effects of operating variables such as the influences of aqueous pH, contact time, Cu(II) concentration, and doses of RHC4 on adsorption were studied. The maximum adsorption was achieved at 120 min of contact time, pH 6, and at 8 g/L of RHC4 dose. The prediction of percentage Cu(II) adsorption was investigated via an artificial neural network (ANN). The Fletcher–Reeves conjugate gradient backpropagation (BP) algorithm was the best fit among all of the tested algorithms (mean squared error (MSE) of 3.84 and *R^2^* of 0.989). The pseudo-second-order kinetic model fitted well with the experimental data, thus indicating chemical adsorption. The intraparticle analysis showed that the adsorption process proceeded by boundary layer adsorption initially and by intraparticle diffusion at the later stage. The Langmuir and Freundlich isotherm models interpreted well the adsorption capacity and intensity. The thermodynamic parameters indicated that the adsorption of Cu(II) by RHC4 was spontaneous. The RHC4 adsorption capacity is comparable to other agricultural material-based adsorbents, making RHC4 competent for Cu(II) removal from wastewater.

## 1. Introduction

Increasing industrial growth has led to the constant accumulation of potential toxic metals at an exponential rate into the environment, which is a major threat to all forms of life. Various industrial effluents discharged normally contain potential toxic metals. They are naturally persistent, toxic, and bio-accumulative, affecting the dynamics of the food web [1]. 

Cu(II) is a micronutrient that is needed for fetal brain development and maintaining the brain’s health throughout life. It is normally found in the human body (between 50 mg to 120 mg), vital for the production of billions of protein molecules, particularly cofactors for enzymes. However, a high dose of Cu(II) is extremely toxic [2]. It has been reported that a Cu(II) dose of more than 1.3 mg/L can cause liver toxicity, jaundice, stomach and intestine diseases, and neurotoxicity [3,4]. A Cu(II) concentration of more than 5 mg/L may lead to kidney failure, high body temperature, rupturing of red blood cells, and nausea [5]. 

Various wastewater treatment technologies have been employed for potential toxic metal removal, such as electrochemical, ion exchange, precipitation, reverse osmosis, membrane filtration, and adsorption [6,7]. Among these technologies, adsorption is an attractive technique due to the ease in handling, low cost, and no sludge production [8,9,10].

Industries preferably practice the adsorption technique using activated carbon for the treatment of potential toxic metal contaminated effluents and other pollutants due to its high removal efficiency and capability to regenerate. Worldwide consumption of commercial activated carbon for wastewater treatment has increased extensively [11]. In industrialized countries, commercial activated carbon use for water treatment accounts for more than 70% of the total demand [12]. However, commercially available activated carbons are generally overpriced, leading to extensive research for cheaper alternative adsorbents. Adsorbents derived from low-cost materials such as peanut husk [13], sugarcane cellulose [14], coconut husk [15], *Lonicera japonica* [16], fly ash [17], carob shells [18], waste newspapers [19], and banana peels [20] have been previously reported for potential toxic metal removal from aqueous solutions. Similarly, rice husk can be a forerunner in the wastewater treatment industry. Agricultural biowaste is an abundant offshoot in Malaysia that can produce an alternative adsorbent for Cu(II) removal. The physicochemical characteristics of rice husk such as water resistance (insolubility), chemical stability, and physically high structural strength make it a good adsorbent. Moreover, cellulose, hemicellulose, and lignin are the major contents in rice husk that contain functional groups, which can be possible adsorption sites [8,21]. However, some modifications, thermal or chemical, need to be carried out before rice husk can be effectively applied as an adsorbent. In this study, rice husk modification was carried out by thermal treatment, and a low-cost rice husk char (RHC4) was developed as an alternative to high-cost activated carbons.

Process modeling and optimization are vital steps to enhance system performance at no additional cost. An artificial neural network (ANN) is a multivariate tool that is used to optimize a system. It is a reliable and powerful tool that models the trajectories of multiple species or variables with an assumption that variables are from normal distribution or free of complexities [22,23]. An ANN has been used in the modeling of chemical reactions in various areas of environmental engineering. However, very few studies have reported its application in heavy metal adsorption. 

The objective of this research was to optimize Cu(II) adsorption in an aqueous solution using rice husk char (RHC4) via ANN modeling. 

## 2. Results and Discussion

### 2.1. Physicochemical Characteristics of RHC4

**Figure 1:**
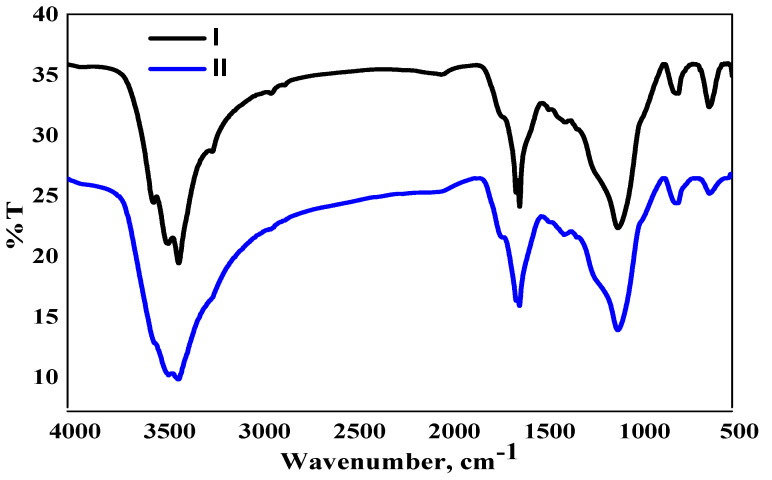
Fourier transform infrared spectroscopy (FTIR) of (I) rice husk char (RHC4) and (II) Cu(II)-loaded RHC4 (adsorbent dose: 8 g/L, Cu(II) concentration: 80 mg/L, contact time: 120 min).

Fourier transform infrared spectroscopy (FTIR) was used to determine the key functional groups on the surface of the adsorbents, namely RHC4 (control) and Cu(II)-loaded RHC4 (Figure 1). Weak peaks were observed at 3545 cm^−1^ and 2031 cm^−1^ representing hydroxyl (O-H) stretching and stretching vibration of nitrile (C≡N), respectively [24,25]. Changes occurred in the vibrational frequencies of functional groups during the adsorption process. These functional groups disappeared after Cu(II) was adsorbed on RHC4 showing their active role as adsorption sites. The wide peak at 1100 cm^−1^ referred to the siloxane (Si-O-Si) group [26]. The intensity became lower for Cu(II)-loaded RHC4 and thus shows contribution in adsorption. Two sharp peaks at 620 cm^−1^ and 573 cm^−1^ referred to alkyl halides and the deformation of the silicon monoxide (Si-O) group, respectively [27]. Based on these observations, the silica and carbon functional groups on the surface of RHC4 played an important role as active Cu(II) adsorption sites.

**Figure 2:**
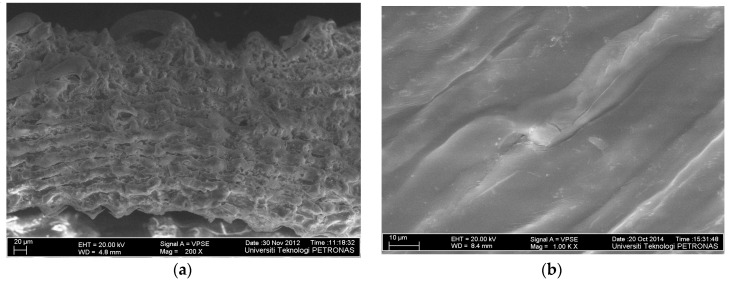
Scanning electron microscopy (SEM) images of (**a**) RHC4 and (**b**) Cu(II)-loaded RHC4 (adsorbent dose: 8 g/L, Cu(II) concentration: 80 mg/L, contact time: 120 min).

Field-emission scanning electron microscopy (FESEM) images of RHC4 and Cu(II)-loaded RHC4 are shown in Figure 2. RHC4 consisted of a rough surface with many pores, where there was good Cu(II) adsorption possibility (Figure 2a). The surface of Cu(II)-loaded RHC4 became smooth, showing the Cu(II) ions’ adherence onto the surface (Figure 2b). Moreover, many pores were found to be absent. It indicated that empty pores were occupied by Cu(II) ions and, hence, adsorption took place. 

A carbon, hydrogen, nitrogen, and sulfur (CHNS) analyzer was used to obtain the elemental data of RHC4 and the analysis revealed that RHC4 was composed of 34.15% carbon, 3.13% hydrogen, 3.54% nitrogen, and 0.162% sulfur. Thus, the presence of sulfur and carbon in RHC4 makes it a good adsorbent [28].

The ash content and bulk density of RHC4 were 18% and 0.2 g/mL, respectively. Other physicochemical properties of RHC4 were Brunauer–Emmett–Teller (BET) surface area (76.47 m^2^/g), micropore area (14.29 m^2^/g), micropore volume (0.0069 mL/g), average pore diameter (40.20 Å), pH_ZPC_ (3.3), and pH (4.18), which was measured by a method used by Ahmedna, et al. [29]. A suspension of RHC4 (1% wt/wt) in deionized water was prepared. The suspension was heated to 90 °C and stirred for 20 min. After cooling to room temperature, the pH of the suspension was measured by a precalibrated pH meter.

### 2.2. Algorithms

A total of eight backpropagation (BP) training algorithms were tested to determine the best fit training algorithm (46 experimental sets) for the prediction of Cu(II) adsorption efficiency by varying the number of neurons in the range of 4 to 40. The applicability of different BP algorithms for the prediction of Cu(II) adsorption is compared in Table 1. It was found that the Fletcher–Reeves conjugate gradient BP algorithm was the most suitable for predicting adsorption of Cu(II) as indicated by the lowest mean squared error (MSE) of 3.84 and highest *R^2^* of 0.989. 

### 2.3. Aqueous pH Influence on Cu(II) Adsorption

**Figure 3:**
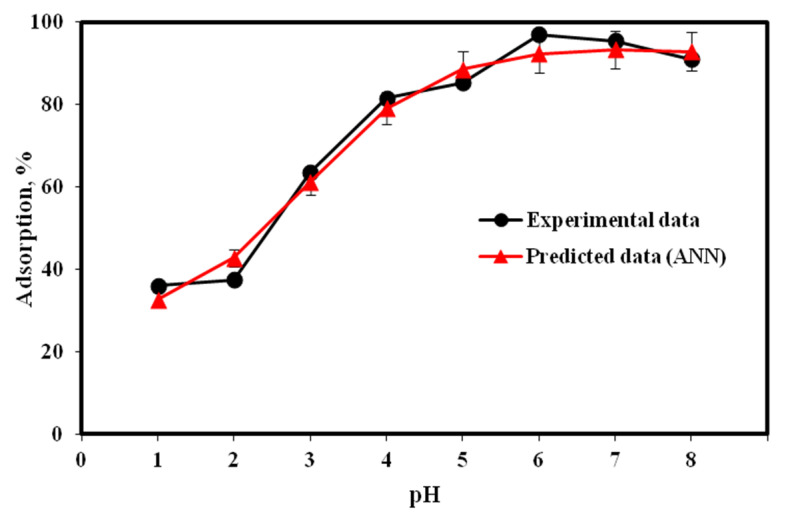
Aqueous pH influence on Cu(II) adsorption (adsorbent dose: 2 g/L, Cu(II) concentration: 20 mg/L, contact time: 120 min, temperature: 22 °C, volume of solution: 100 mL).

The influence of aqueous pH on adsorption efficiency is shown in Figure 3. The adsorbent’s surface charge and degree of metal ionization in solution were aqueous pH dependent. An increase in aqueous pH increased the adsorption efficiency to 97.07% at pH 6. At low pH, adsorbent became positively charged due to the high concentration of protons (H^+^) present in the solution. This scenario resulted in the protonation of some surface functional groups, thereby repelling Cu(II) ions entering into the RHC4 pores or even approaching the RHC4 surface. Meanwhile, at higher pH, protons were removed from the adsorbent due to deprotonation and neutralized by the hydroxyl ions (OH^−^), thus increasing the adsorption of Cu(II) on the RHC4 surface. Moreover, the concentration of negatively charged surface functional groups increases when pH increases, allowing covalent bonding among these groups and the cationic heavy metals [30].

The behavior and capability of an adsorbent to adsorb contaminants from a solution can be explained in terms of pH of the zero point of charge (pH_ZPC_). As reported by Mall, et al. [31], cation adsorption is favored at a pH greater than pH_ZPC_, whereas anion adsorption is favored at a pH less than pH_ZPC_. The obtained pH_ZPC_ value of RHC4 was 3.3. As shown in Figure 3, the maximum Cu(II) adsorption onto RHC4 was achieved at pH 6, which is higher than the value of pH_ZPC_. Similar trends have been reported for Cu(II) adsorption using other adsorbents such as coconut dregs residue [32] and garden grass [33]. Overall, the ANN model satisfactorily predicted the trend of the experimental data.

### 2.4. Influence of Cu(II) Concentration and Contact Time

Contact time is a key parameter that affects the adsorption process. Both the adsorbent and adsorbate should be in contact for enough time to attain maximum adsorption. The influence of Cu(II) concentration and contact time on adsorption efficiencies is shown in Figure S1 (Supplementary Materials). The adsorption efficiency decreased with an increase in Cu(II) concentration irrespective of contact time. During the initial stage of adsorption, a large number of vacant surface sites were available for adsorption. After some time, the remaining vacant surface sites were not easily occupied due to repulsive forces between the solute molecules on the solid surface and the bulk phase [34]. Meanwhile, a lower concentration of Cu(II) ions would be anticipated to find more binding sites on the adsorbent as compared to higher concentrations, and therefore, facilitate adsorption. The adsorption equilibrium was achieved at 120 min. Altun and Pehlivan [35] and Güzel, et al. [36] reported a similar contact time using an adsorbent developed from walnut shell and pomegranate pulps. Therefore, a contact time of 120 min was used for subsequent experiments along with the modeling via an ANN. The experimental and predicted data produced by the ANN were in good agreement. Thus, an ANN model can precisely predict Cu(II) adsorption onto RHC4 based on the initial Cu(II) concentration and the effect of contact time. 

### 2.5. Influence of RHC4 Dose

**Figure 4:**
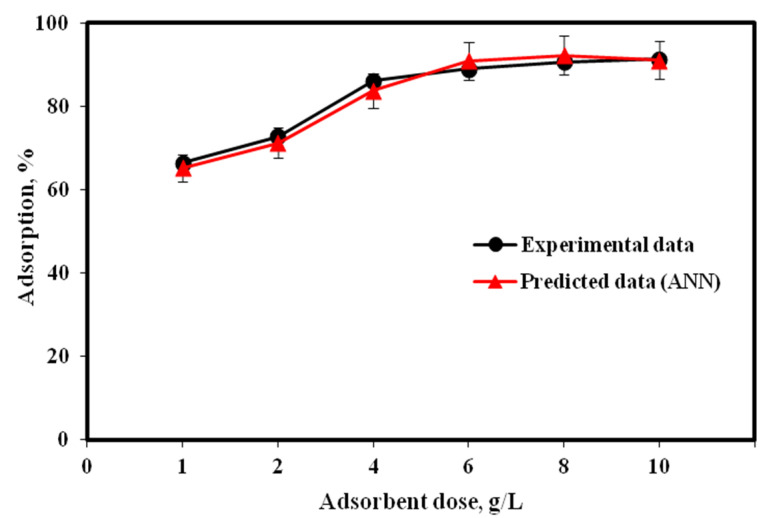
Influence of RHC4 dose on adsorption (Cu(II) concentration: 80 mg/L, contact time: 120 min, temperature: 22 °C, volume of solution: 100 mL).

The influence of RHC4 dose on the adsorption efficiency is shown in Figure 4. The dose of adsorbent was varied from 1 to 10 g/L in a Cu(II) solution (80 mg/L). The other operating variables such as contact time (120 min) and optimum pH (6) remained the same. The adsorption efficiencies increased steadily with an increase in RHC4 dose, due to a larger contact surface of adsorbent particles and more active sites for adsorption [37]. Maximum adsorption efficiency was achieved (90.64%) at 8 g/L RHC4. Overall, the experimental data were in agreement with the ANN predicted model. 

### 2.6. Kinetics

Kinetic models such as pseudo-first-order Equation (1) [38], pseudo-second-order Equation (2) [39], Elovich Equation (3) [40], and intraparticle diffusion models Equation (4) [41] were used to assess Cu(II) adsorption by RHC4 over time (Figure S2, Supplementary Materials). The linear forms of these kinetics models are presented below:

(1) $$\log(q_{e}-q_{t})=\log q_{e}-\frac{k_{1}t}{2.303}$$

(2) $$\frac{t}{q_{t}}=\left(\frac{1}{k_{2}q_{e}^{2}}\right)+\left(\frac{t}{q_{e}}\right)$$

(3) $$q_{t}=\frac{1}{\beta }\ln\left(\alpha \beta \right)+\frac{1}{\beta }\;\ln\left(t\right)$$

(4) qt=kpt12+C

where *q_e_* and *q_t_* are the quantities (mg/g) of Cu(II) adsorbed at equilibrium and at any time *t*, respectively; *k*_1_ (min^−1^) and *k*_2_ [g/(mg min)] are the reaction rate constants for pseudo-first-order and pseudo-second-order kinetic models, respectively; *α* is the initial adsorption rate (mg/g min); *β* is related to the extent of surface coverage and activation energy involved in chemisorption (g/mg); *k_p_* is a measure of diffusion coefficient (mg/g min^1/2^); and *C* represents the thickness of the adsorption boundary layer (mg/g).

The values of calculated rate constants and *R*^2^ are presented in Table 2. The pseudo-first-order model showed significant difference between *q_e,exp_* and *q_e,cal_*. The *R*^2^ values for pseudo-second-order were equivalent to 0.99 with approximately 1% difference between *q_e,exp_* and *q_e,cal_* values. The values of *q_e_* increased from 9.51 mg/g to 29.11 mg/g for 20 mg/L and 80 mg/L Cu(II) concentration, respectively. In addition, it was observed that the variation of the rate constant, *k*_2_, seems to have a decreasing trend for increasing Cu(II) concentration The sum of squared error (SSE) results between the experimental data and the predicted values were minimized using the solver add-in function of Microsoft Excel [42] and are also presented in Table 2. A comparison of the SSE of these kinetic models shows that the pseudo-second-order kinetic model fitted better. Hence, it can be concluded that the adsorption of Cu(II) by RHC4 followed the pseudo-second-order kinetic model, strongly suggesting chemical adsorption. The kinetic constants derived from the Elovich equation are also listed in Table 2. It can be seen that the values of both *α* and *β* decreased with an increase in Cu(II) concentration from 20 to 80 mg/L.

The values of intraparticle diffusion constant *C* (boundary layer thickness) increased with an increase in Cu(II) concentration (Table 2). Thus, the resistance to the external mass transfer increases as the Cu(II) concentration increases [43]. A similar trend has also been observed by Bandura et al. [44] and Hossain et al. [33] for Cu(II) adsorption using synthetic zeolite and garden grass, respectively. 

### 2.7. Isotherms 

Isotherm models such as the Langmuir Equation (5), Freundlich Equation (6), and Sips (combined Langmuir–Freundlich expression) Equation (7) models show the degree of adsorbate accumulation on any adsorbent surface at constant temperature [45,46]. The Langmuir, Freundlich, and Sips [47] adsorption isotherms are expressed in the following equations:

(5) $$q_{e}=\frac{Q^{o}bC_{e}}{1+bC_{e}^{}}$$

where *Q^o^* is the sum of the adsorbed solute on the surface of the adsorbent that forms a monolayer (monolayer adsorption capacity) and *b* is the energy of adsorption.

(6) $$q_{e}=K_{f}C_{e}{}^{1/n}$$

where *K_f_* is the Freundlich constant and 1/*n* is the adsorption intensity.

(7) $$q_{e}=\frac{q_{m}b_{s}C_{e}^{\frac{1}{n}}\;}{{(1+b}_{s}C_{e}^{\frac{1}{n}})\;}$$

where *q*_e_ is the maximum adsorption capacity of the RHC4 adsorbent and *b_s_* is the energy of adsorption.

**Figure 5:**
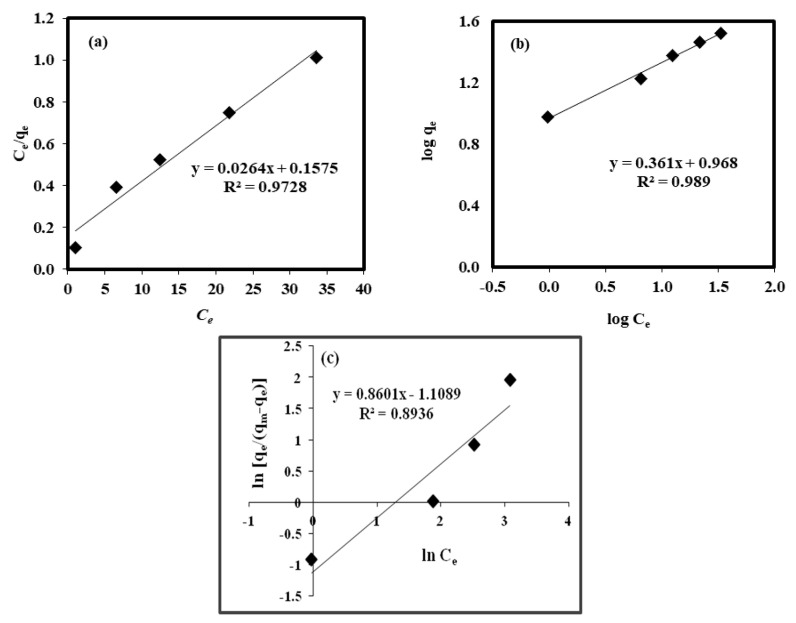
Linearized (**a**) Langmuir isotherm, (**b**) Freundlich isotherm, and (**c**) Sips models (adsorbent dose: 2 g/L, Cu(II) concentration: 20, 40, 60, 80, and 100 mg/L, contact time: 120 min, temperature: 22 °C, volume of solution: 100 mL).

By considering the optimum pH and contact time, adsorption isotherms were developed for multiple Cu(II) concentrations. The adsorption isotherm data were fitted with linear forms of the Langmuir isotherm (*C_e_/q_e_ = 1/(bQ^o^) + C_e_/Q^o^*) (Figure 5a), the Freundlich isotherm (*log q_e_ = log K_f_ + (*1*/n) log C_e_*) (Figure 5b), and the Sips isotherm (*ln (q_e_/q_m_ − q_e_) = *1*/n(C_e_) + ln(b_s_)^*1*/n^* (Figure 5c). The values of *Q^o^* and *b* (Langmuir constants), *K_f_* and 1/*n* (Freundlich constants), and *b_s_* and 1/*n* (Sips constants) are listed in Table 3. The *R*^2^ values were 0.97 (Langmuir), 0.98 (Freundlich), and 0.89 (Sips). The high *R*^2^ value (0.98) and the lowest SSE value of 0.02 (Table 3) simulated by the Freundlich isotherm demonstrate that adsorption can be explained by a multilayer adsorption mechanism and occurs on heterogeneous surfaces [48]. This heterogeneity can be attributed to various interactions of RHC4 and functional groups on the surface of RHC4. Moreover, the value of *1*/*n* is less than 1, indicating a good adsorption effect. 

The distinctiveness of the Langmuir isotherm can be expressed by a dimensionless constant, *R_L_* (equilibrium parameter) [49,50] Equation (8):

(8) $$R_{L}=\frac{1}{1+bC_{o}}$$

In this equation, *b* represents the Langmuir constant and *C_o_* is the initial adsorbate concentration (Cu(II)). From the values of *R_L_*, it can be concluded whether the isotherm is unfavorable (*R_L_* > 1), linear (*R_L_* = 1), favorable (0 < *R_L_* < 1), or irreversible (*R_L_* = 0). The values of *b* (Table 3) for Cu(II) concentrations ranging between 20 mg/L to 100 mg/L showed that the adsorption of Cu(II) onto RHC4 is favorable (*R_L_* lies between 0 and 1).

### 2.8. Influence of Temperature and Thermodynamic Parameters 

It is essential to take into consideration the energy and entropy in order to determine whether an adsorption process will proceed spontaneously. The practical application of the adsorption process mainly depends on the values derived from thermodynamic parameters. The effect of temperature on adsorption and its mechanism were investigated by varying the temperature from 25 °C to 60 °C and the values of the thermodynamic parameters, i.e., change in free energy (∆*G^o^*), enthalpy (∆*H^o^*), and entropy (∆*S^o^*) were calculated from the following equations:

(9) $$K_{C}=\frac{C_{A}}{C_{S}}$$

(10) $$\Delta G^{o}=-RT\ln K_{C}$$

(11) $$\ln K_{C}=\frac{\Delta S^{o}}{R}-\frac{\Delta H^{o}}{RT}$$

where:

*K_C_* = equilibrium constant

*C_A_* = sum of adsorbed Cu(II) onto RHC4 (mg/L)

*C_S_* = Cu(II) concentration in solution at equilibrium (mg/L)

*R* = 8.314 J mol^−1^ K^−1^

*T* = working temperature (*K*)

∆*G^o^* = Gibbs free energy

∆*H^o^* = slope from the plot of *ln K_C_* versus 1/*T*

∆*S^o^* = intercept from the plot of *ln K_C_* versus 1/*T*

**Figure 6:**
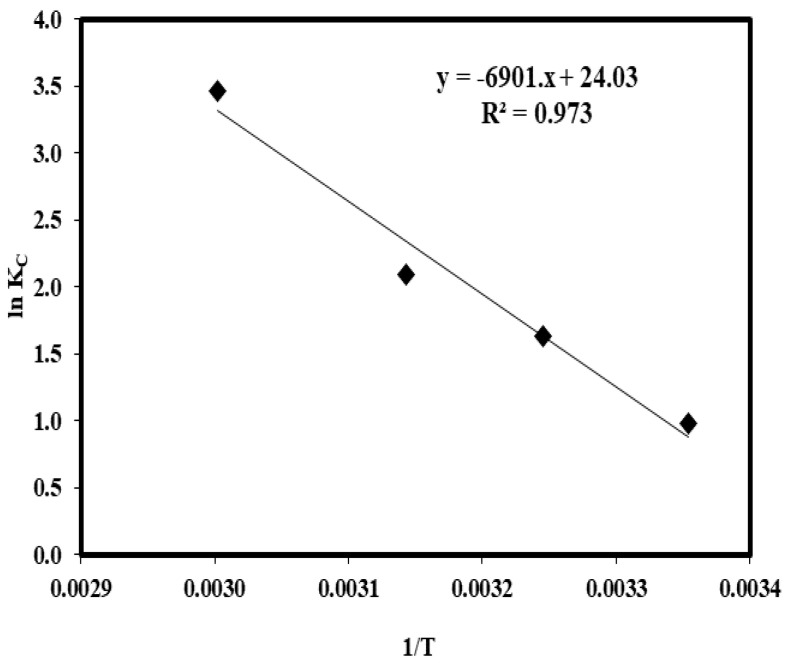
Plot of *ln K_C_* vs. 1/*T*.

The thermodynamic plot (*ln K_C_* versus 1/*T*) is shown in Figure 6 and the values of the thermodynamic parameters are given in Table 4. The Gibbs free energy values are negative. This is an indication of a spontaneous and feasible adsorption mechanism. A positive value of ∆*H^o^* (57.37 kJ/mol) projected the adsorption as endothermic. The ∆*S^o^* was 199.78 J/mol. It referred to an increase in the randomness of solid–solution interface activity, during Cu(II) adsorption onto RHC4 [51]. The higher the temperature, the more efficient the adsorption process becomes, as evidenced with the increase in the adsorption capacity (*q_e_*) with temperature.

### 2.9. Cu(II) Adsorption Efficiency of Different Types of Adsorbents

The Cu(II) adsorption capacity of different adsorbents derived from agricultural by-products and their surface areas reported by other researchers are summarized in Table 5. Compared with other adsorbents, RHC4 indicated excellent efficiency for Cu(II) adsorption. Thus, RHC4 can be commercialized as a substitute to commercial activated carbon for the removal of Cu(II) and other heavy metal ions from water and wastewater. Further investigation on the adsorption efficiency of other heavy metals is highly recommended.

## 3. Materials and Methods 

### 3.1. Development and Physicochemical Properties of RHC4

Rice husk was obtained from a rice paddy processing factory in Perak Tengah, Malaysia. The collected rice husk was rinsed several times with tap and distilled water for dust removal followed by oven-drying (105 °C) overnight. Next, the rice husk was incinerated using a muffle furnace (Nabertherm, Bahnhofstr, Germany) (300 °C) for 4 h to produce the adsorbent, namely rice husk char (RHC4). The produced RHC4 was milled into powder (212 μm to 500 μm) and used in multiple batch adsorption tests.

The physicochemical properties of RHC4 were determined using FTIR (SHIMADZU, Kyoto, Japan), FESEM (Zeiss, Oberkochen, Germany), a CHNS analyzer (LECO, St. Joseph MI, USA), BET surface area (Micrometrics, Norcross, GA, USA) analysis, bulk density (g/mL), ash content (%), pH, and pH_ZPC_. 

### 3.2. Adsorption Experiment

The working solutions (1000 mg/L) were prepared by dilution of a copper nitrate (Cu(NO_3_)_2_ 3H_2_O) stock. The working solutions’ initial pH values were altered with 0.1 N HCL and NaOH solutions. Batch adsorption tests were conducted in conical flasks (150 mL) with 100 mL Cu(II) aqueous solutions, then adsorbents were added and stirred using an orbital shaker (150 rpm). The ranges of pH (pH 1 to pH 8), contact time (5 min to 180 min), initial Cu(II) concentration (20 mg/L to 80 mg/L), and adsorbent dose (1 g/L to 10 g/L) were studied. The adsorbents were filtered using a membrane filter (0.45 µm). The Cu(II) concentration was determined using an atomic absorption spectrophotometer (SHIMADZU, Kyoto, Japan). 

### 3.3. Modeling

**Figure 7:**
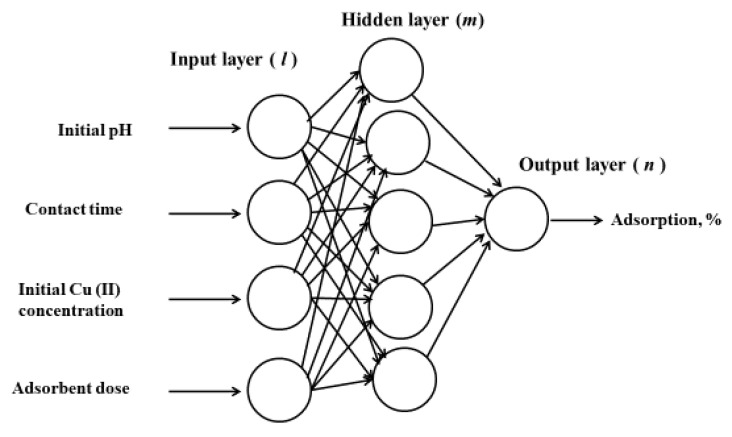
Artificial neural network (ANN) architecture.

The ANN modeling trails numerical prediction techniques resembling the human brain and nervous system mechanism [20]. Moreover, it is fully functional on nonlinear relationships [62], making it a powerful alternative for adsorption system prediction. An ANN learns from experiences or examples (i.e., actual input data and corresponding outputs) in order to decide the set of rules that controls the relationship among the variable species [63]. An ANN is composed of input, hidden, and output layers. The basic unit is called a neuron that has summing and weight functions. The summing function sums the output values, whereas the weight function enrolls the logic part, a nonlinear function [64], as shown in Figure 7. 

A three-layered backpropagation neural network was used with a neural transfer function (purelin) at the output layer and a tangent sigmoid transfer function (tansig) at the hidden layer. A total of 46 experimental sets was developed in the ANN model (MATLAB R2013a, Natick, MA, USA) consisting of input [p] and target [t] matrices. The pH, Cu(II) concentration, contact time, and RHC4 dose were tabulated as inputs. The Cu(II) adsorption (%) was identified as target response. The training (70%), validation (15%), and testing (15%) groups were accommodated with 30, 8, and 8 data sets, respectively.

## 4. Conclusions

The conclusions from this study are summarized as follows:

The FTIR study showed silica and carbon, functional groups, on the surface of RHC4 that played an active role in the adsorption of Cu(II).

The best-fit backpropagation algorithm for the prediction of Cu(II) removal from an aqueous solution using RHC4 was the Fletcher–Reeves conjugate gradient (MSE of 3.84 and *R*^2^ of 0.989). 

Optimum Cu(II) adsorption was achieved at contact time 120 min, pH 6, and 8 g/L RHC4 dose.

The best kinetic model was pseudo-second-order, indicating chemical adsorption. The adsorption process proceeded by surface or boundary layer adsorption at the initial stage and by intraparticle diffusion at the final stage.

Langmuir constants *Q^o^* and *b* were 38.46 and 0.16, Freundlich constants *K_f_* and 1/*n* were 9.28 and 0.36, and Sips constants *b_s_* and 1/*n* were 0.27 and 0.86, respectively. 

Thermodynamic values indicated that the adsorption of Cu(II) by RHC4 is a spontaneous and feasible process in the range of experimental temperatures studied.

The Cu(II) adsorption capacity of RHC4 was higher than many low-cost adsorbents reported in the literature. 

## Figures and Tables

**Table 1:** Backpropagation training algorithms. MSE, mean squared error.

Algorithm	Function	Optimal Neuron Number	MSE	*R* ^2^
Resilient	*trainrp*	16	57.48	0.908
Fletcher–Reeves conjugate gradient	*traincgf*	10	3.84	0.989
Polak–Ribière–Polyak conjugate gradient	*traincgp*	10	4.78	0.986
Powell–Beale conjugate gradient	*traincgb*	10	3.88	0.988
Levenberg–Marquardt	*trainlm*	6	4.50	0.987
Scaled conjugate gradient	*trainscg*	10	6.79	0.981
BFGS quasi-Newton	*trainbfg*	18	7.83	0.980
One-step secant	*trainoss*	8	7.07	0.979

**Table 2:** Kinetics parameters.

Model	Parameters	Cu(II) Concentration			
		20 mg/L	40 mg/L	60 mg/L	80 mg/L
	*q_e_,_exp_* (mg g^−1^)	9.51	16.73	23.76	29.11
Pseudo-first-order	*q_e,cal_* (mg g^−1^)	1.1	2.1	3.73	4.98
	*k*_1_ (min^−1^)	0.02	0.023	0.016
	*R* ^2^	0.97	0.98	0.98	0.97
	*SSE*	8.41	14.63	20.03	24.13
Pseudo-second-order	*q_e_*,*_cal_* (mg g^−1^)	9.61	16.94	24.39	29.41
	*k*_2_ (g mg^−1^ min^−1^)	0.071	0.038	0.016	0.012
	*R* ^2^	0.99	0.99	0.99	0.99
	*SSE*	0.1	0.21	0.63	0.3
Elovich	*α* (mg/g min)	233,279	82,015	58,965	29,128
	*β* (g/mg)	2.89	1.54	0.97	0.67
	*R* ^2^	0.98	0.99	0.92	0.96
	*SSE*	1.09	0.64	0.77	0.78
Intraparticle diffusion	*k_p_* (mg g^−1^ min^−1/2^)	0.12	0.22	0.38	0.53
	*C* (mg g^−1^ )	8.23	14.35	19.47	23.12
	*R* ^2^	0.969	0.95	0.99	0.98
	*SSE*	1.28	2.38	4.29	5.99

**Table 3:** Values of Langmuir and Freundlich constants.

Isotherm	Constants		*R* ^2^	*SSE*
Langmuir	*Q^o^* (mg/g)	*b* (L/g)	0.97	0.50
	38.46	0.16		
Freundlich	*K_f_* (mg/g)	1/*n*	0.98	0.02
	9.28	0.36		
Sips	*b_s_*	1/*n*	0.89	0.69
	0.27	0.86		

**Table 4:** Thermodynamic parameters.

T (°C)	*q_e_* (mg/g)	*K_C_*	∆*G^o^*	∆*H^o^* (kJ/mol)	∆*S^o^* (J/mol)
25 °C	29.11	2.64	−2.44	57.37	199.78
35 °C	33.48	5.14	−4.19		
45 °C	35.62	8.14	−5.55		
60 °C	38.78	31.92	−9.59		

**Table 5:** Comparison of Cu(II) adsorption efficiencies of different adsorbents.

Adsorbent	Surface Area (m^2^/g)	Adsorption Capacity (mg/g)	Reference
Raw pomegranate peel	598.78	30.12	[1]
Grape bagasse activated carbon	1455	37.17	[52]
Palm oil fruit shell	39.76	20–60	[53]
Banana peel	2.0	20.97	[54]
Pineapple peel fiber	-	27.68	[55]
Pine cone powder	-	26.23	[56]
Irish peat moss	203.41	17.6	[57]
Hazelnut husk	4.31	6.645	[58]
Ceiba pentandra hulls	521	20.8	[59]
Cellulose pulp waste	2.64	4.98	[60]
Compost	1.36	12.77	[60]
Tree fern	2.39	11.7	[61]
Rice husk char	76.47	38.46	This study

## Supplementary Materials

The following are available online. Figure S1: The influence of initial Cu(II) concentration and contact time on adsorption. Figure S2: Kinetic models: (**a**) pseudo-first-order kinetic plot, (**b**) pseudo-second-order kinetic plot, (**c**) Elovich and (**d**) intraparticle diffusion plot of Cu(II) adsorption by RHC4.
