# Supplementary material for: Modeling of Cu(II) Adsorption from an Aqueous Solution Using an Artificial Neural Network (ANN)
Source: Molecules. 2020 Jul 17;25(14):3263. doi: 10.3390/molecules25143263 (PMC7397182; doi:10.3390/molecules25143263)
Supplement: Supplementary file 1 [file molecules-25-03263-s001.pdf]

molecules

SUPPLEMENTARY MATERIALS

Modeling of Cu(II) Adsorption from an Aqueous Solution Using an Artificial Neural Network (ANN)

Taimur Khan [1,2,*], Teh Sabariah Binti Abd Manan [3], Mohamed Hasnain Isa [4], Abdulnoor A.J. Ghanim [1], Salmia Beddu [5], Hisyam Jusoh [6], Muhammad Shahid Iqbal [7], Gebiaw T Ayele [8] and Mohammed Saedi Jami [9]

1 Department of Civil Engineering, Faculty of Engineering, Najran University, P.O Box 1988, King Abdulaziz Road, Najran, Saudi Arabia; aaghanim@nu.edu.sa
2 Civil and Environmental Engineering Department, Universiti Teknologi PETRONAS, 32610 Seri Iskandar, Perak Darul Ridzuan, Malaysia
3 Institute of Tropical Biodiversity and Sustainable Development, Universiti Malaysia Terengganu, Kuala Terengganu 21300, Malaysia; tehsabariah@umt.edu.my
4 Civil Engineering Programme, Universiti Teknologi Brunei, Tungku Highway, Gadong BE1410, Brunei Darussalam; mohamed.isa@utb.edu.bn
5 Department of Civil Engineering, Universiti Tenaga Nasional, Jalan Ikram-Uniten, 43000 Kajang, Selangor, Darul Ehsan, Malaysia; salmia@uniten.edu.my
6 Geo TriTech, No. 17, Persiaran Perdana 15A, Pinji Perdana, Lahat 31500, Perak, Malaysia; mnhisyam.jusoh@gmail.com
7 Department of Space Sciences, Institute of Space Technology, Islamabad 44000, Pakistan; muhammad.shahidiqbal@outlook.com
8 Australian Rivers Institute and School of Engineering, Griffith University, Nathan, QLD 4111, Australia; gebeyaw21@gmail.com
9 Department of Biotechnology Engineering, Faculty of Engineering, International Islamic University Malaysia, P.O. Box 10, Kuala Lumpur 50728, Malaysia; saedi@iium.edu.my
* Correspondence: taimurkhan7@mail.com or tkkhan@nu.edu.sa; Tel: +966-590643452

SUPPLEMENTARY MATERIALS CONTENT

Figure S1. The influence of initial Cu(II) concentration and contact time on adsorption.

Figure S2. Kinetic models: (**a**) pseudo-first-order kinetic plot, (**b**) pseudo second order, (**c**) Elovich, and (**d**) intraparticle diffusion plot of Cu(II) adsorption byRHC4.

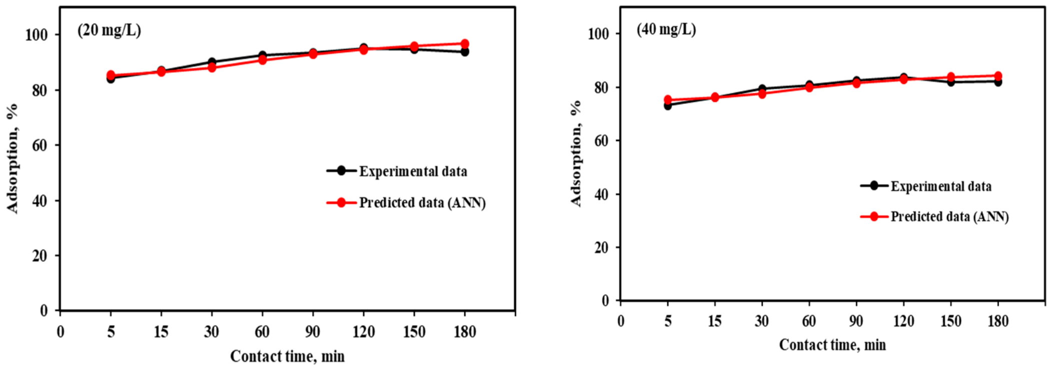

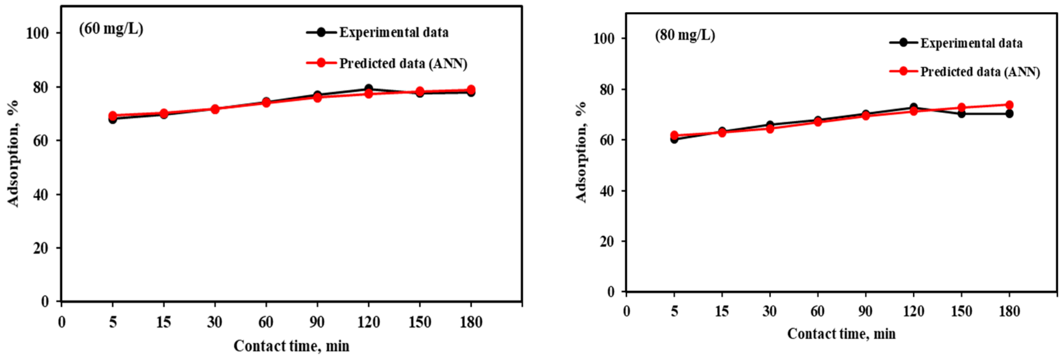

Figure S1. The influence of initial Cu(II) concentration and contact time on adsorption (Adsorbent dose: 2g/L, contact time: 180 min, temperature: 22 °C, volume of solution: 100 mL).

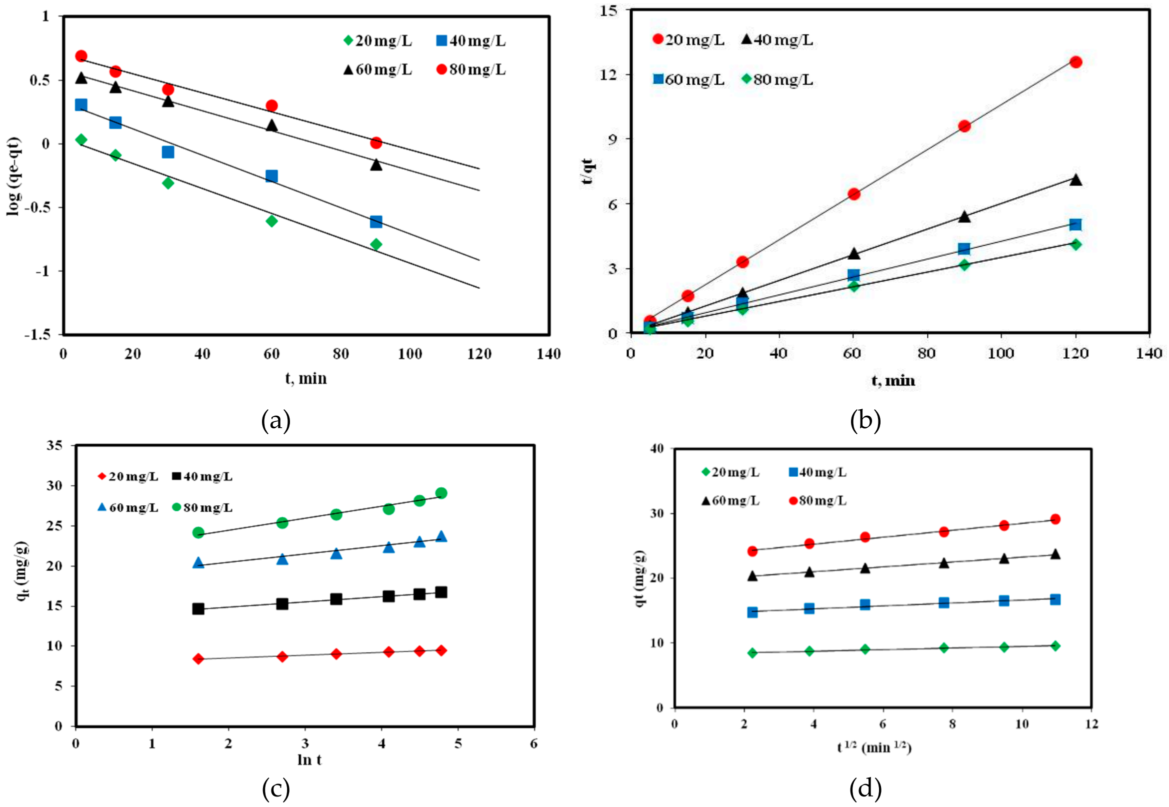

Figure S2. Kinetic models: (**a**) pseudo-first-order kinetic plot, (**b**) pseudo second order, (**c**) Elovich and (**d**) intraparticle diffusion plot of Cu(II) adsorption by RHC4 (Adsorbent dose: 2g/L, contact time: 120 min, temperature: 22 °C, volume of solution: 100 mL).

© 2020 by the authors. Licensee MDPI, Basel, Switzerland. This article is an open access article distributed under the terms and conditions of the Creative Commons Attribution (CC BY) license (http://creativecommons.org/licenses/by/4.0/).
